# Non-invasive molecular imaging of inflammatory macrophages in allograft rejection

**DOI:** 10.1186/s13550-015-0146-7

**Published:** 2015-11-26

**Authors:** Alexander S. G. O’Neill, Samantha Y. A. Terry, Kathryn Brown, Lucy Meader, Andrew M. S. Wong, Jonathan D. Cooper, Paul R. Crocker, Wilson Wong, Gregory E. D. Mullen

**Affiliations:** Department of Imaging Chemistry and Biology, Division of Imaging Sciences and Biomedical Engineering, King’s College London, St. Thomas’ Hospital, London, SE1 7EH UK; Division of Medical Sciences, University of Oxford, John Radcliffe Hospital, Oxford, OX3 9DU UK; MRC Centre for Transplantation, King’s College London, Guy’s Hospital, London, UK; Pediatric Storage Disorders Laboratory, Department of Neuroscience and Centre for the Cellular Basis of Behaviour, King’s College London, London, UK; Division of Cell Signalling and Immunology, College of Life Sciences, University of Dundee, Dundee, UK

**Keywords:** Macrophages, Cardiac transplantation, Preclinical imaging, Sialoadhesin, SER-4

## Abstract

**Background:**

Macrophages represent a critical cell type in host defense, development and homeostasis. The ability to image non-invasively pro-inflammatory macrophage infiltrate into a transplanted organ may provide an additional tool for the monitoring of the immune response of the recipient against the donor graft. We therefore decided to image in vivo sialoadhesin (Sn, Siglec 1 or CD169) using anti-Sn mAb (SER-4) directly radiolabelled with ^99m^Tc pertechnetate.

**Methods:**

We used a heterotopic heart transplantation model where allogeneic or syngeneic heart grafts were transplanted into the abdomen of recipients. In vivo nanosingle-photon emission computed tomography (SPECT/CT) imaging was performed 7 days post transplantation followed by biodistribution and histology.

**Results:**

In wild-type mice, the majority of ^99m^Tc-SER-4 monoclonal antibody cleared from the blood with a half-life of 167 min and was located predominantly on Sn^+^ tissues in the spleen, liver and bone marrow. The biodistribution in the transplantation experiments confirmed data derived from the non-invasive SPECT/CT images, with significantly higher levels of ^99m^Tc-SER-4 observed in allogeneic grafts (9.4 (±2.7) %ID/g) compared to syngeneic grafts (4.3 (±10.3) %ID/g) (*p* = 0.0022) or in mice which received allogeneic grafts injected with ^99m^Tc-IgG isotype control (5.9 (±0.6) %ID/g) (*p* = 0.0185). The transplanted heart to blood ratio was also significantly higher in recipients with allogeneic grafts receiving ^99m^Tc-SER-4 as compared to recipients with syngeneic grafts (*p* = 0.000004) or recipients with allogeneic grafts receiving ^99m^Tc-IgG isotype (*p* = 0.000002).

**Conclusions:**

Here, we demonstrate that imaging of Sn^+^ macrophages in inflammation may provide an important additional and non-invasive tool for the monitoring of the pathophysiology of cellular immunity in a transplant model.

**Electronic supplementary material:**

The online version of this article (doi:10.1186/s13550-015-0146-7) contains supplementary material, which is available to authorized users.

## Background

Macrophages are tissue-resident components of the innate and adaptive immune systems and perform a variety of functions in host defense and maintenance of homeostasis [1]. As such, they are crucial in the progress and resolution of a variety of pathological conditions, including cancer, autoimmunity, atherosclerosis and rejection of transplanted organs [2]. There are a variety of antigens used in the ex vivo identification of human macrophages, and of these, the markers CD64, CD68 (Macrosialin), CD163, CD169 (sialoadhesin) and CD204 (macrophage scavenger receptor A) represent the set of *trans*-species pan-macrophage markers [3]. Due to the significant plasticity of macrophage phenotype, producing a single macrophage marker has eluded researchers and none is uniquely expressed on macrophages. CD64 is expressed on monocytes and subsets of germinal and blood dendritic cells, CD68 on various leukocytes, CD163 on monocytes, CD204 on monocytes and dendritic cells, and of the most myeloid restricted of these (CD163) significant amounts are found as soluble product [4], complicating its use for in vivo non-invasive imaging.

To date, the majority of macrophage imaging has been performed by magnetic resonance imaging (MRI) using non-specific nanoparticles such as superparamagnetic iron oxide (SPIO). SPIOs were injected intravenously (i.v.) and taken up in vivo by phagocytic cells [5]. This uptake is not unique to macrophages and cells such as dendritic cells can also take up iron oxide particles [6]. Other approaches have involved ex vivo non-specific labelling of macrophages with a contrast agent, such as a nanoparticle or a radiolabel, followed by MRI or single-photon emission computed tomography imaging (SPECT) imaging [7]. Recently, the targeting of macrophages with labelled antibodies has begun to be explored, with a number of groups reporting success with this technology in preclinical models. ^111^In-labelled anti-F4/80-A3-1 [8], ^68^Ga-labelled CD163 [9] and optically labelled CD206 [10] have shown the feasibility and utility of macrophage targeting in vivo*.* In addition, radiotracers targeting translocator protein (TSPO) as a biomarker of microglial activation and macrophage infiltration in the brain have been used [11].

Here, we report non-invasive in vivo imaging specific for inflammatory macrophages using the anti-sialoadhesin (Sn, Siglec 1 or CD169) monoclonal antibody, SER-4 [12]. Increasing attention is being paid towards the marker Sn [13, 14], which under quiescent conditions is expressed on subsets of macrophages in secondary lymphoid tissues, such as the lymph nodes and spleen [12]. However, Sn^+^ macrophages can also be found in a variety of pathological conditions [15–17]. Sn^+^ macrophages not only exhibit classic innate immune cell behaviour by acting as professional phagocytes but also display a close relation in promoting immune responses [18] through the activation of other immune effector cells including CD8 T cells [19], B cells [20] and iNKT cells [21]. This relationship is demonstrated by enhanced immunity resulting from the targeting of antigenic material to Sn^+^ macrophages [22, 23] and also by the amelioration of autoimmunity following Sn knock-down [24–26]. Increasingly, Sn expression is being linked clinically with disease progression in a variety of settings and is finding use as a marker of inflammation [27].

There is still a clinical necessity for further development of non-invasive imaging biomarkers not only for the diagnosis and staging of disease but also for interim assessment of therapies. Solid organ transplantation is one area where the development of a non-invasive imaging biomarker would aid therapy response assessment. The incidence of acute transplant rejection within the first year has decreased dramatically by the introduction of modern immunosuppressive therapies, while the rates of chronic transplant rejection have remained high [28]. While efforts are underway for the non-invasive imaging of ischemia reperfusion injury post transplantation [29], not much has been done in the way of non-invasive imaging of recipient macrophages in graft rejection. Thus, close surveillance of transplanted organs remains imperative. The current clinical standard of repetitive invasive endomyocardial biopsies is prone to sampling error, entails a risk of severe complications, causes discomfort and anxiety for the patients and, unlike for kidney transplants, is usually performed as a last resort. Therefore, developing non-invasive yet quantitative diagnostic tools for the monitoring of allograft rejection would fulfil an unmet clinical need.

The aim of this study is to test the biodistribution of ^99m^Tc-SER-4 in normal animals and an inflammatory model such as an acute rejection model.

## Methods

### Mice, culture media, reagents and antibodies

C57BL/6 (H-2b) and BALB/c (H-2d) mice were ordered from Harlan Olac (Bicester, UK). Sn knockout (Sn^-/-^) mice were bred and maintained in the Biological Services Unit at King’s College London. RPMI 1640 medium (Sigma, Poole, UK), supplemented with 5 mM L-Glut (Invitrogen, Paisley, UK), 100 U/mL penicillin (Invitrogen), 100 μg/mL streptomycin (Invitrogen), 10 % IgG-depleted foetal calf serum (Source Bioscience UK Ltd., Nottingham, UK), 1 mM Hepes (Invitrogen) and 0.05 mM mercaptoethanol (Invitrogen), was used for antibody production, labelling and in vitro binding assays. Antibodies were purified using 5 mL HiTrap Protein G HP and 13.5 mL G-25 Sephadex desalting columns (PD-10) (GE Healthcare, Chalfont St. Giles, UK). Size exclusion chromatography (SEC) was performed using an Agilent 1200 series (Agilent, Oxford, UK) high-performance liquid chromatography (HPLC) system with in-line UV (280 nm) and radionuclide detector (Flow-Count, LabLogic, UK).

### Purification and technetium-99 m radiolabeling of SER-4 antibody

Anti-mouse Sn SER-4 antibody was isolated as previously described using the SER-4 hybridoma [12]. Briefly, SER-4 hybridoma cells were grown in Celline CL350 (Integra Biosciences AG, Zissers, Switzerland) according to manufacturer’s instructions. Tissue culture media was then harvested and purified on a protein G column followed by dialysis into PBS (Gibco). The SER-4 and the anti-mouse IgG isotype control (AbD Serotec, Oxon, UK) antibodies were directly radiolabelled with ^99m^Tc. Briefly, antibodies were concentrated to 10 mg/mL, using a Vivaspin 20 centrifugal concentrator (Sartorius Stedim, Epsom, UK), and 2 mg (200 μL, 13 nM) was then reduced by a molar excess of 2-mercaptoethanol (2-ME, 2000:1, 2 μl, 26 μM) at room temperature for 30 min. The reduced antibody was purified using a PD-10 desalting column and stored in PBS at −80 °C at 5 mg/mL. For antibody radiolabeling, 5 μl of a reconstituted MDP kit (Medronate Draximage, Draxis, USA) was added to 0.1 mg (20 μL, 0.67 nM) of reduced SER-4, followed by the addition of 150 MBq of sodium pertechnetate (provided by Department of Nuclear Medicine at Guys Hospital, UK). Labelling efficiency was measured using instant thin layer chromatography strips (ITLC-SA) (Varian Medical Systems UK, Ltd., Crawley, UK) with a mobile phase of 0.1 M citrate buffer, pH 5 and analysed using a gamma ray TLC scanner (Lablogic, UK). The amount of colloids has not been assessed but that large colloids anyway would have been eliminated by filtration prior to injection.

### Stability assay

Fifty MBq of ^99m^Tc SER-4 was added to AB type human serum (Sigma) or PBS at 1:4 *v*/*v* and incubated at 37 °C for 20 h. Samples were analysed at 0, 3, 6 and 20 h by HPLC-SEC using a BioSep SEC-300 column (Phenomenex, Macclesfield, UK) with an isocratic mobile phase of 100 mM phosphate buffer pH 7.0 at a flow rate of 1 mL/min. Stability was calculated as the area under the ^99m^Tc-SER-4 peak (retention time = 8 min and 30 s) versus the area under the curve of the unbound ^99m^Tc peak (retention time = 18 min).

### In vitro ^99m^Tc SER-4 binding assay

Of labelled antibody, 0.1 μg was added to 0.2 μg of recombinant Sn-Fc fusion protein (Sn-Fc, 6.7 μg/ml) and incubated at 37 °C for 10 min. The proteins were filtered through a 0.2-μm syringe filter and binding to Sn-Fc was measured by HPLC-SEC and compared to the ^99m^Tc-IgG isotype binding. Specificity was shown in blocking studies using a 10-fold excess of cold SER-4. No Kd or immunoreactive fraction measurements were performed; as the authors felt that the in vitro competitive binding assay was sufficient to proceed to preclinical studies.

### NanoSPECT/CT transplant imaging

Heterotopic cardiac transplantations were performed on 8- to 10-week-old male C57BL/6 mice (three groups, *n* = 5 for SER4 allogeneic and isotype allogeneic groups) with heart graft from BALB/c mice while syngeneic cardiac transplants (*n* = 4 for SER4 syngeneic group) with heart grafts from C57BL/6 mice as described by Corry et al [30]. All mice were imaged and ex vivo biodistribution was performed. Briefly, superior and inferior vena cava and pulmonary veins of the heart graft were ligated. The donor aorta was then anastomosed to the recipient abdominal aorta and the donor pulmonary artery anastomosed to the inferior vena cava, resulting in a fully vascularised heterotopic transplant. C57BL/6, Sn-deficient (Sn^-/-^) and transplanted mice were anesthetized with inhaled isoflurane gas (VetOne, UK) and ~10 μg (~20 MBq) of ^99m^Tc-SER-4 or ^99m^Tc-IgG isotype control was filtered through a 0.2-μm syringe filter and administered intravenously. Single-photon emission computed tomography (SPECT) images were obtained 3 h post injection using a nanoSPECT/computed tomography (CT) preclinical scanner (Bioscan Inc., Washington, DC, USA) equipped with four heads, each with 1-mm multipinhole collimator, in helical scanning mode in 24 projections over 30 min. The CT images were obtained with 45 kVP X-ray source, 1000 ms exposure time in 180 projections over 10 min. Images were reconstructed in a 256 by 256 matrix using the HiSPECT (Scivis GmBH) reconstruction software package and fused using InVivoScope software (Bioscan). Images shown here are maximum intensity projections (MIP). Animals were then euthanized at 4 h post injection and tissues explanted, weighed and gamma counted on a gamma counter (Wallac, 1282 Compugamma, PerkinElmer, UK). Uptake in each tissue was expressed as percent injected dose per gram of tissue (%ID/g). The transplanted heart to blood ratio was calculated by expressing the %ID/g of the transplanted heart divided by %ID/g in the blood. After imaging, C57BL/6 wild-type and Sn^-/-^ spleens or grafted hearts and spleens from C57BL/6 recipients were removed for histology which was performed as previously described [12].

### Immunostaining

After imaging and biodistribution, frozen tissues were stained for Sn using SER-4 followed by anti-rat-biotin then streptavidin-HRP. Sn^+^ macrophages were visualized with Vector NovaRED substrate (red) and sections counterstained with haemotoxylin.

### Statistical analysis

To test for a significant differences between ^99m^Tc-SER-4 in allogeneic and syngeneic transplants with ^99m^Tc-IgG in allogeneic heart transplants, a one-way ANOVA was first performed; *p* values of <0.05 were considered significant. If the one-way ANOVA was significant, then a post hoc analysis was performed with Student–Newman–Keuls pairwise comparison; *p* values of <0.05 were considered significant.

## Results

### ^99m^Tc-SER-4 is stable in serum and binds to sialoadhesin

Anti-Sn antibody was produced using the SER-4 hybridoma. SER-4 antibody was reduced with mercaptoethanol and directly radiolabelled with sodium pertechnetate in the presence of methylene-diphosphonate ligand. The radiolabelling efficiency as measured by ITLC was >99 % with a specific activity of 1.5 MBq/μg of antibody. ^99m^Tc-SER-4 remained stable in serum for 20 h with only one radioactive peak observed at a retention time of 8 min (see Additional file 1). However, when either the ^99m^Tc-SER-4 or ^99m^Tc-IgG was incubated in PBS alone, free pertechnetate was observed after 20 h (see Additional file 1).

**Fig. 1:**
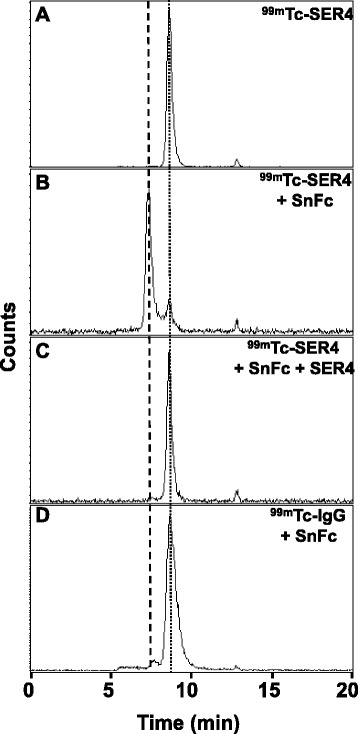
^99m^Tc-SER-4 binds to Sn. Binding studies were performed by incubating ^99m^Tc-SER-4 and Sn-Fc followed by analysis with radio-HPLC size exclusion chromatography. **a** ^99m^Tc-SER-4 elutes at 8 min and 35 s. **b** ^99m^Tc-SER-4 was added to Sn-Fc and an elution peak at 7 min and 20 s was observed corresponding to the ^99m^Tc-SER-4 bound to Sn-Fc. **c** When ^99m^Tc-SER-4 was added to Sn-Fc protein in the presence of unlabelled SER-4, no binding was observed and only the ^99m^Tc-SER-4 elution peak was observed. **d** ^99m^Tc-IgG isotype control did not bind to Sn-Fc with a peak elution time similar to ^99m^Tc-SER-4 of 8 min and 35 s

To ensure that the radiolabelling procedure did not adversely affect the ability of SER-4 to bind to Sn, an in vitro binding assay was developed. Binding was determined by observing a shift in retention time from 8 min and 35 s of unbound ^99m^Tc-SER-4 (Fig. 1a) to ^99m^Tc-SER-4 bound to Sn-Fc with a retention time of 7 min and 20 s (Fig. 1b). The binding of ^99m^Tc-SER-4 bound to Sn-Fc was inhibited by the addition of unlabelled SER-4 antibody (Fig. 1c). The ^99m^Tc-IgG isotype control did not bind to Sn-Fc and eluted with a retention time of 8 min and 30 s similar to that of unbound ^99m^Tc-SER-4 (Fig. 1d).

We did not run an SDS-PAGE to show that the antibody remains intact as the radiolabeled antibody is still able to bind >99 % to Sn antigen which is completely blocked by cold antigen in a competitive binding assay.

### Biodistribution of ^99m^Tc-SER-4 in mice

**Fig. 2:**
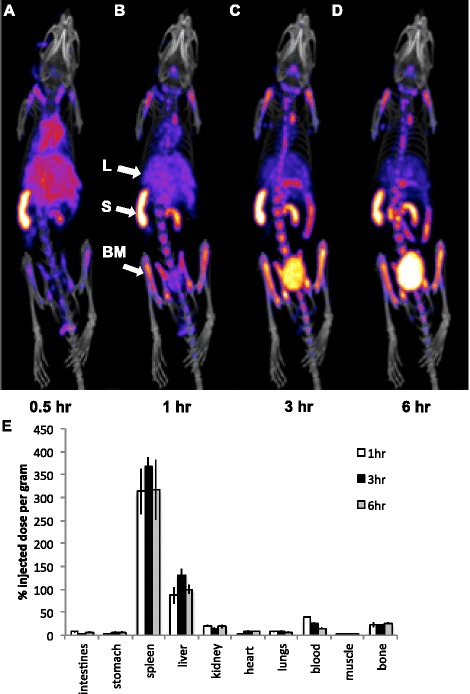
^99m^Tc-SER-4 NanoSPECT/CT imaging and biodistribution of Sn^+^ macrophages. C57Bl/6 wild-type (WT) mice were imaged **a** 0.5 h, **b** 1 h, **c** 3 h and **d** 6 h post injection with ^99m^Tc-SER-4. *Arrows* indicate uptake of tracer by Sn^+^ macrophages in the spleen (*S*), liver (*L*) and bone marrow (*BM*). After imaging, C57BL/6 wild-type (WT) mice were euthanized and tissues explanted, weighed and gamma counted. Maximum intensity projections are shown. **e** ^99m^Tc-SER-4 biodistributions at 1, 3 and 6 h post injection were expressed as percentage injected dose per gram of tissue (%ID/g). *Error bars* represent the standard error of the mean of *n* = 3

C57BL/6 wild-type mice, injected with 20 MBq of ^99m^Tc-SER-4, were imaged at 0.5, 1, 3 and 6 h post injection (Fig. 2). At 1 h, ^99m^Tc-SER-4 uptake was already observed in the spleen, liver and bone marrow, which were the expected locations of Sn^+^ macrophages. Additionally, bladder uptake is consistent with previous studies of directly labelled antibodies and is thought to result from transchelation of ^99m^Tc from disulphides to endogenous cysteine [31]. It has been previously shown that antibodies labelled via the direct labelling method which were then challenged with cysteine led to ~10 % translocation of the Tc-99 m to the cysteine [32].

Blood levels of ^99m^Tc-SER-4 continued to drop between 3 and 6 h. After imaging, mice were euthanized and standard radioactive biodistribution was performed. The biodistribution results confirmed the non-invasive SPECT/CT images (Fig. 2e). ^99m^Tc-SER-4 clears the blood rapidly with 66.7 %ID/g in the blood immediately post injection to 15.0 ± 0.9 %ID/g at 6 h with a clearance half-life in blood of 167 min (*p* = 0.0004). This is significantly faster blood clearance than expected for most radiolabelled monoclonal antibodies.

**Fig. 3:**
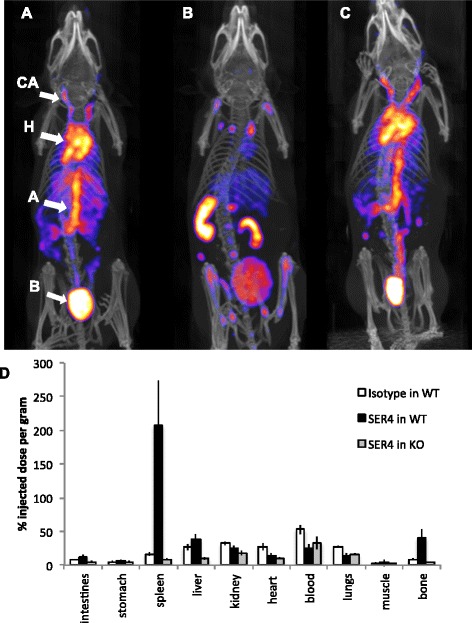
^99m^Tc-IgG and ^99m^Tc-SER-4 nanoSPECT/CT imaging and biodistribution in C57Bl/6 wild-type (*WT*) and Sn^-/-^ (KO) mice. Imaging was performed 3 h post injection on C57Bl/6 WT mice with **a** ^99m^Tc-IgG isotype control or **b** ^99m^Tc-SER-4 or **c** Sn^-/-^ mice with ^99m^Tc-SER-4*. Arrows* indicate signal arising from carotid arteries (*CA*), heart (*H*), aorta (*A*) and bladder (*B*). Maximum intensity projections are shown. **d** Biodistribution of ^99m^Tc-SER-4 in C57Bl/6 wild-type, Sn^-/-^ and ^99m^Tc-IgG isotype control in C57Bl/6 wild-type mice were expressed as percentage injected dose per gram of tissue (%ID/g). *Error bars* represent the standard error of the mean of *n* = 5

As a control, ^99m^Tc-SER-4 or ^99m^Tc-IgG isotype was injected into Sn^-/-^ or C57BL/6 wild-type mice, respectively, and imaged by nanoSPECT/CT at 3 h post injection (Fig. 3). Both ^99m^Tc-SER-4 and ^99m^Tc-IgG remained in the blood of Sn^-/-^ or wild-type mice, respectively, and reflected the typical biodistribution expected of a 150-kDa radiolabelled monoclonal antibody. Biodistribution results confirmed the SPECT/CT images where the majority of the ^99m^Tc-SER-4 (200 ± 66.0 %ID/g) was observed in the spleen of wild-type mice. Splenic uptake was lower for ^99m^Tc-SER-4 (8.3 ± 1.1 %ID/g) or ^99m^Tc-IgG (16.5 ± 2.6 %ID/g) in Sn^-/-^ or wild-type mice, respectively (*p* = 0.0029 and 0.0025, respectively). The majority of ^99m^Tc-IgG (52.9 ± 7.0 %ID/g) in wild-type mice was retained in the blood as compared to ^99m^Tc-SER-4 in wild-type mice (26.1 ± 5.4 %ID/g) (Fig. 3d) (*p* = 0.002). Staining of spleen sections from wild-type mice with SER-4 gave the distinctive stain of Sn^+^ marginal metallophilic macrophages (MMM) in the internal border of the white pulp (Additional file 2). Raw biodistribution data for blood clearance and controls are available in supplemental data (Additional files 3 and 4).

### Non-invasive imaging of ^99m^Tc-SER-4 targets Sn^+^ macrophages in heterotopic cardiac transplantations

**Fig. 4:**
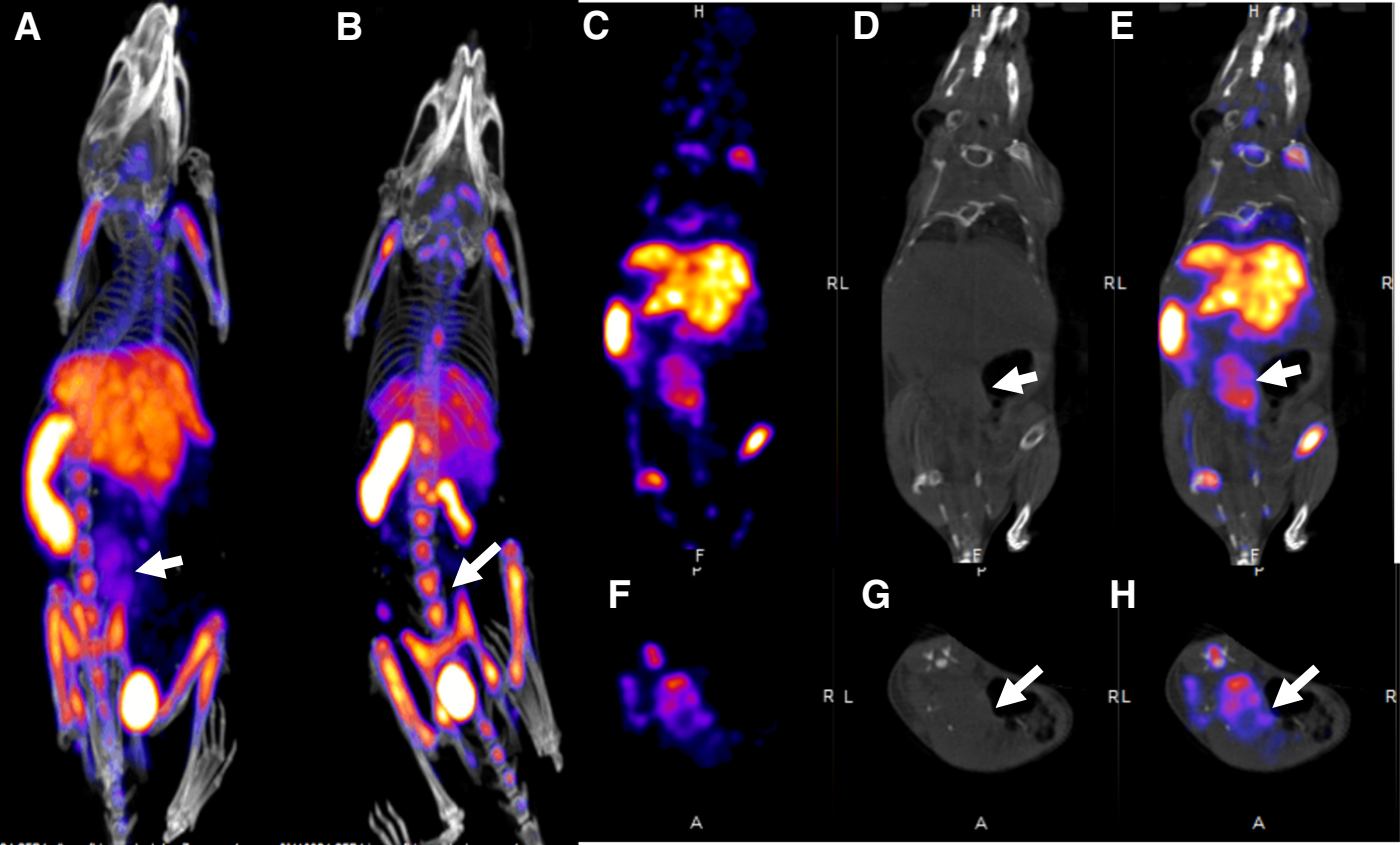
^99m^Tc-SER-4 nanoSPECT/CT imaging of Sn^+^ macrophages in heterotopic cardiac transplant model. Representative maximum intensity projection nanoSPECT/CT images of recipients with either **a** allogeneic or **b** syngeneic heart graft imaged 3 h post injection with ^99m^Tc-SER-4. CT, SPECT and fused images from coronal (**c**, **d**, **e**, respectively) and transversal (**f**, **g**, **h**, respectively) planes are also shown. *Arrows* indicate site of abdominal cardiac transplant with uptake of tracer in allogeneic heart graft that are absent in the syngeneic heart graft model

Seven days after transplantation of allogeneic or syngeneic heart grafts into the abdomen, recipients underwent nanoSPECT/CT imaging at 3 h post injection of ^99m^Tc-SER-4 (Fig. 4). Uptake was observed in the allogeneic graft in the abdomen of recipients (Fig. 4a) but not in recipients receiving a syngeneic graft (Fig. 4b). Again, high levels of ^99m^Tc-SER-4 were observed in the spleen, liver and bone marrow with only low levels of uptake in non-target tissues.

**Fig. 5:**
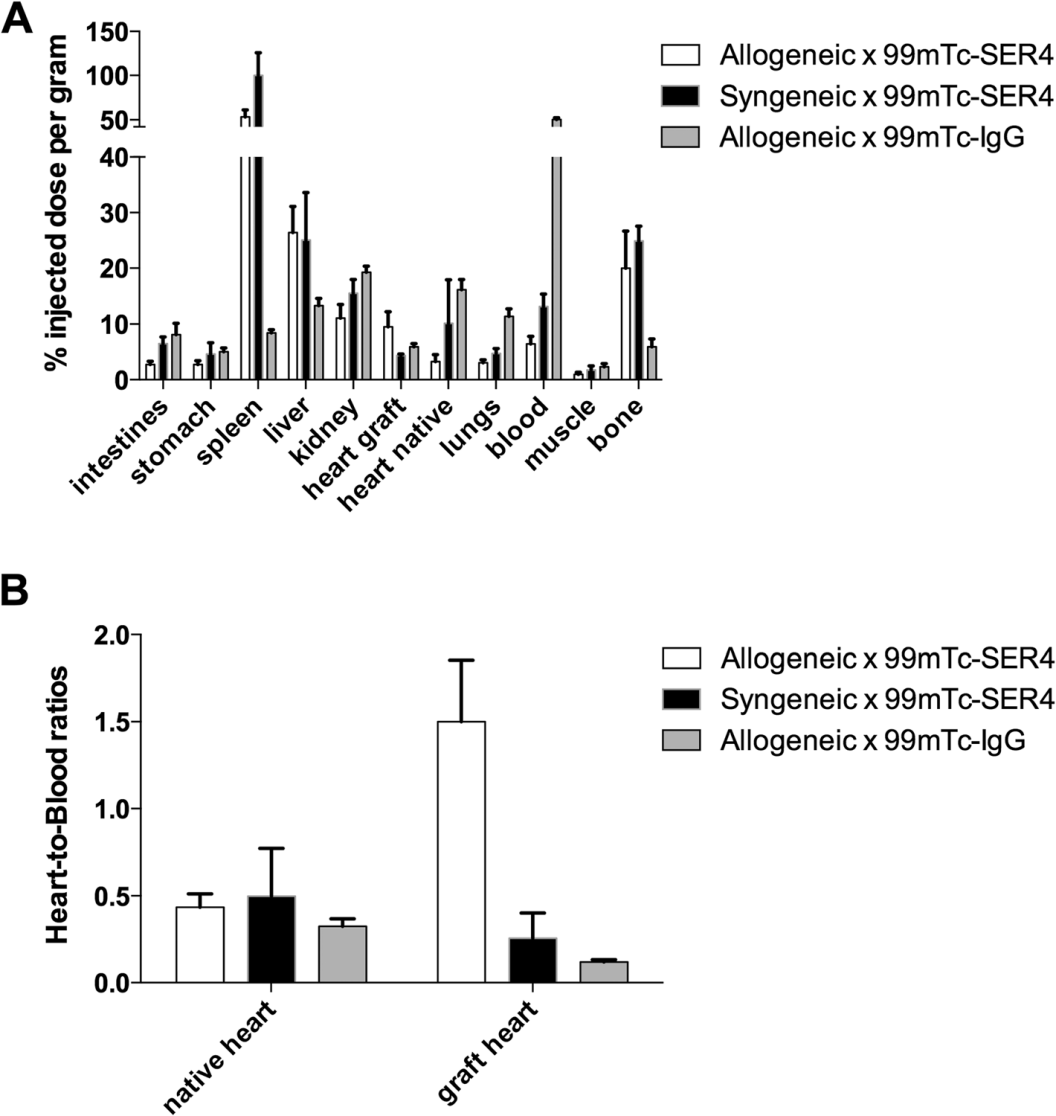
Biodistribution of ^99m^Tc-SER-4 in heterotopic cardiac transplant model. **a** Biodistribution of ^99m^Tc-SER-4 in recipients of allogeneic heart graft, syngeneic heart grafts or allogeneic heart grafts at 4 h post injection of ^99m^Tc-IgG isotype control. **b** Transplanted heart to blood ratios for ^99m^Tc-SER-4 in recipients of allogeneic heart graft, syngeneic heart grafts or allogeneic heart grafts injected with ^99m^Tc-IgG isotype control. *Error bars* represent the standard error of the mean of *n* = 4–5

Biodistribution data confirmed a significantly higher uptake of ^99m^Tc-SER-4 in the allogeneic heart grafts (9.4 ± 2.7 %ID/g) compared to syngeneic heart graft (4.3.0 ± 0.3 %ID/g) (*p* = 0.0022) or in mice which received allogeneic grafts and injected with ^99m^Tc-IgG isotype control (5.9 ± 0.6 % ID/g) (*p* = 0.0185) (Fig. 4). Given that 50.3 ± 2.2 %ID/g of the ^99m^Tc-IgG isotype control was observed at 4 h in the blood, transplanted heart to blood ratios were also calculated. The heart to blood ratio was significantly higher in recipients with allogeneic grafts receiving ^99m^Tc-SER-4 as compared to recipients with syngeneic grafts (*p* = 0.000004) or recipients with allogeneic grafts receiving ^99m^Tc-IgG isotype (*p* = 0.000002) (Fig. 5). Raw biodistribution data for transplanted mice is available in supplemental data (Additional file 5).

**Fig. 6:**
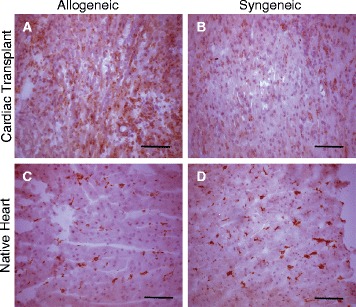
Cardiac histology. Histology of heterotopic cardiac sections from **a** allogeneic grafts and **b** syngeneic grafts are shown. Controls were performed by staining of both **c** allogeneic and **d** syngeneic recipient heart tissue. Images are shown at ×20 magnification (scale bar = 100 μm)

The presence of Sn^+^ macrophages in allogeneic grafts was confirmed in transplants stained with anti-Sn. Allogeneic grafts showed an abundance of Sn^+^ macrophages while sporadic expression of Sn^+^ macrophages were observed in syngeneic grafts and minor staining in recipient’s native hearts (Fig. 6).

## Discussion

We have been able to perform non-invasive imaging specific for macrophages using a radiolabelled antibody. Although much is known about the phenotype of macrophages, these have not been exploited for the development of non-invasive imaging contrast agents. Monoclonal antibodies are being successfully used in immunotherapy of diseases such as cancer where they target antigens such as HER2 using trastuzumab (Herceptin), EGFR with panitumumab (Vectibix) and VEGF-A with bevacizumab (Avastin). Antibodies targeted specifically at immune cells, such as granulocytes, B and T cells, have also been evaluated as diagnostic contrast agents for the imaging of infection, rheumatoid arthritis and transplant rejection [33]. However, to date, few antibodies are used routinely for diagnostic imaging and many of these have been withdrawn from the market or are still under clinical development [34].

With macrophages being implicated, for better or worse, in many diseases, it is important to be able to target these cells non-invasively to assess prognosis as well as interim assessment of therapeutic interventions. We therefore decided to radiolabel one of the most restricted macrophage surface markers sialoadhesin (CD169). Low expression of Sn was observed in many tissue macrophages (such as in the liver, dermis and lung) but significant expression was observed in macrophages in the secondary lymphoid organs and bone marrow [12]. Sn expression can be upregulated in inflammation via inflammatory mediators such as TNF-α and type I IFN in humans, rats and pigs [15, 35, 36]. SER-4 was radiolabelled with the radioisotope ^99m^Tc(^99m^Tc-SER-4) and found to be stable in serum but lower stability was observed in PBS after 20 h. We then performed in vivo imaging and biodistribution studies in wild-type mice and in a murine model of heterotopic cardiac transplant. The majority of ^99m^Tc-SER-4 monoclonal antibodies was cleared from the blood within 3 h and was located in predominately Sn^+^ M tissues such as the spleen, liver and bone marrow. ^99m^Tc-SER-4 uptake was not observed in these tissues in Sn^-/-^ mice, and ^99m^Tc-IgG isotype control remained in the blood for the duration of the experiment. In the heterotopic cardiac transplants, it was possible to observe ^99m^Tc-SER-4 in allogeneic heart grafts but not in syngeneic heart grafts. This was further quantified by the biodistribution studies which showed significant uptake of ^99m^Tc-SER-4 as compared to radiolabelled isotype control or syngeneic. Histology of the transplanted grafts also demonstrated the presence of Sn^+^ macrophages in the allogeneic heart grafts, and only sporadic expression was observed in the syngeneic or native hearts.

Conventional wisdom tells us that monoclonal antibodies are too large for fast real-time in vivo imaging as they take several days to clear from the blood and also penetrate tissue slowly. However, if one is able to clear non-targeted radiolabelled antibodies quickly from circulation by either providing a fast excretion route via the kidney or liver (which is unlikely for large antibodies) or an endogenous “sink” (identified by biodistribution to be predominantly bone marrow, liver and spleen), then significant targeting of regions of interests may be possible even with low target to background ratios. We have previously also seen evidence of this sink when using antibodies targeting macrophages at 24 h post injection [8]. To note, we did see non-specific uptake in the kidney using 99mTc-SER-4 biodistribution data which may question its suitability for imaging macrophage infiltration in kidney transplants.

More recent studies have indicated a role for macrophage-mediated rejection as a contributing factor for the progressive decline in graft function, indicating that current immunosuppressive treatment protocols fail to keep at bay the potent effector responses of the adaptive immune system [37]. This imaging technique could equally be used in imaging a model of chronic rejection, and as with acute rejection, the current view is that macrophages promote worse graft outcome through the release of inflammatory mediators and regulation of the cytokine dynamics [38]. Macrophages are a major component of inflammatory infiltrates in rejecting allografts [39]. Macrophages are rapidly recruited to sites of inflammation including allografts where they are responsive to type I interferons, while at the same time potent producers of pro-inflammatory cytokines such as IL-1, IL-6 and TNF-beta [40]. The early infiltration of macrophages post organ transplantation has been observed in biopsies demonstrating acute cellular rejection as well as in acute humoral rejection and has been shown to be associated with relatively poor allograft survival [41]. More recently, increased monocyte expression of sialoadhesin was observed during acute cellular rejection after intestine transplantation in children [42].

## Conclusions

Radiolabelled SER4 has demonstrated the ability to specifically image non-invasively pro-inflammatory macrophage infiltrate into rejected grafts, which may provide an important additional tool for the monitoring of transplanted organs.

## Additional files

Additional file 1: Figure S1. Serum stability of ^99m^Tc-SER-4. ^99m^Tc-SER-4 was incubated in the presence of phosphate-buffered saline (B) or serum (C) for 20 h at 37 °C then injected into SEC radioHPLC system. Percentage bound was assessed by comparison of bound activity (integral of 8 min and 25 s peak) to that of free activity (integral of 17 min and 45 s peak). ^99m^TcO4 is included as a control (A). (PDF 73 kb) Additional file 2: Figure S2. Spleen sections from wild-type animals stained with SER-4 and counterstained with haematoxylin. White arrow denotes white pulp, black arrow denotes red pulp and black arrowhead denotes marginal metallophilic macrophages. (PDF 798 kb) Additional file 3: Table S1. Timecourse biodistribution of 99mTc-SER-4 in wild-type mice. Biodistribution of ^99m^Tc-SER-4 in wild-type mice at 1, 3 and 6 h post injection. Data expressed as percentage injected dose per gram of tissue (%ID/g). (PDF 63 kb) Additional file 4: Table S2. Biodistribution of ^99m^Tc-IgG and ^99m^Tc-SER-4 in C57Bl/6 wild-type (WT) and Sn^-/-^ (KO) mice. Biodistribution of ^99m^Tc-IgG isotype control in C57Bl/6 wild-type mice (WT × Isotype) ^99m^Tc-SER-4 in C57Bl/6 wild-type (WT × ^99m^Tc-SER4) and Sn^-/-^ mice (Sn KO × 99mTcSER4) were expressed as percentage injected dose per gram of tissue (%ID/g). (PDF 70 kb) Additional file 5: Table S3. Biodistribution of 99mTc-SER-4 in heterotopic cardiac transplant model. Biodistribution of ^99m^Tc-SER-4 in recipients of allogeneic heart graft (mouse 1–5), syngeneic heart grafts (mouse 6–9), or allogeneic heart grafts at 4 h post injection of ^99m^Tc-IgG isotype control (mouse 10–14). Data expressed as percentage injected dose per gram of tissue (%ID/g). (PDF 67 kb)

## Abbreviations

mAb: monoclonal antibody
MRI: magnetic resonance imaging
Sn: sialoadhesin
SPECT: single-photon emission computed tomography
SPIO: superparamagnetic iron oxide
