# Supplementary material for: Non-invasive molecular imaging of inflammatory macrophages in allograft rejection
Source: EJNMMI Res. 2015 Nov 26;5:69. doi: 10.1186/s13550-015-0146-7 (PMC4661159; doi:10.1186/s13550-015-0146-7)
Supplement: Additional file 2: Figure S2. — Spleen sections from wild-type animals stained with SER-4 and counterstained with haematoxylin. White arrow denotes white pulp, black arrow denotes red pulp and black arrowhead denotes marginal metallophilic macrophages. (PDF 798 kb) [file 13550_2015_146_MOESM2_ESM.pdf]

ESM Figure 2

Alexander S. G. O'Neill[1,2], Samantha Y.A. Terry[1], Kathryn Brown[3], Lucy Meader[3], Andrew M.S. Wong[4], Jonathan D. Cooper[4], Paul R. Crocker[45], Wilson Wong[3], Gregory E. D. Mullen[1,3*]

[1]Department of Imaging Chemistry and Biology, Division of Imaging Sciences and Biomedical Engineering, King's College London, St. Thomas' Hospital, London, SE1 7EH, UK
[2]Division of Medical Sciences, University of Oxford, John Radcliffe Hospital, Oxford, OX3 9DU
[3]MRC Centre for Transplantation, King's College London, Guy's Hospital, London, UK
[4]Pediatric Storage Disorders Laboratory, Department of Neuroscience and Centre for the Cellular Basis of Behaviour, King's College London, London, UK
[5]Division of Cell Signalling and Immunology, College of Life Sciences, University of Dundee, Dundee, UK

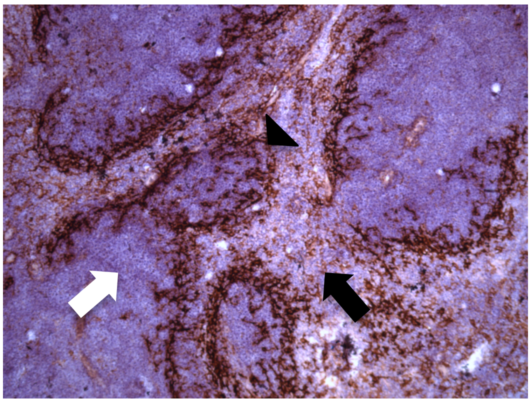

SUPPLEMENTAL FIGURE 2
Spleen sections from with type animals stained with SER-4 and counterstained with haematoxylin. White arrow denotes white pulp, black arrow denotes red pulp, black arrowhead denotes marginal metallophilic macrophages.
