# Supplementary material for: Non-invasive molecular imaging of inflammatory macrophages in allograft rejection
Source: EJNMMI Res. 2015 Nov 26;5:69. doi: 10.1186/s13550-015-0146-7 (PMC4661159; doi:10.1186/s13550-015-0146-7)
Supplement: Additional file 3: Table S1. — Timecourse biodistribution of 99mTc-SER-4 in wild-type mice. Biodistribution of 99mTc-SER-4 in wild-type mice at 1, 3 and 6 h post injection. Data expressed as percentage injected dose per gram of tissue (%ID/g). (PDF 63 kb) [file 13550_2015_146_MOESM3_ESM.pdf]

ESM Table 1

Alexander S. G. O'Neill[1,2], Samantha Y.A. Terry[1], Kathryn Brown[3], Lucy Meader[3], Andrew M.S. Wong[4], Jonathan D. Cooper[4], Paul R. Crocker[45], Wilson Wong[3], Gregory E. D. Mullen[1,3*]

[1]Department of Imaging Chemistry and Biology, Division of Imaging Sciences and Biomedical Engineering, King's College London, St. Thomas' Hospital, London, SE1 7EH, UK
[2]Division of Medical Sciences, University of Oxford, John Radcliffe Hospital, Oxford, OX3 9DU
[3]MRC Centre for Transplantation, King's College London, Guy's Hospital, London, UK
[4]Pediatric Storage Disorders Laboratory, Department of Neuroscience and Centre for the Cellular Basis of Behaviour, King's College London, London, UK
[5]Division of Cell Signalling and Immunology, College of Life Sciences, University of Dundee, Dundee, UK

	1 hr			3 hr			6 hr		
Mouse	1	2	3	4	5	6	7	8	9
intestines	7.14	6.35	9.07	2.99	3.36	3.53	7.37	5.61	6.41
stomach	4.24	2.98	3.68	4.59	7.80	5.24	6.77	5.83	7.77
spleen	352.21	257.18	330.21	379.54	347.95	378.69	390.33	290.06	268.75
liver	100.42	95.52	66.07	116.28	141.65	135.89	93.38	95.13	113.52
kidney	21.94	21.02	20.77	12.38	16.73	13.96	20.82	15.31	19.87
heart	2.31	2.57	2.26	11.04	9.28	9.91	7.80	7.72	9.60
lungs	8.94	7.65	6.55	7.27	10.25	8.85	7.70	4.93	5.75
blood	39.23	37.74	39.76	25.35	27.66	21.19	15.23	13.11	16.73
muscle	0.84	1.27	0.69	0.72	1.02	0.75	0.58	1.36	0.73
bone	16.41	21.89	28.16	24.21	19.56	21.44	25.95	23.85	29.51

ESM Table 1. Timecourse biodistribution of ^{99m}Tc-SER-4 in wild type mice. Biodistribution of ^{99m}Tc-SER-4 in wild type mice at 1 hr, 3 hr and 6 hr post injection. Data expressed as percentage injected dose per gram of tissue (%ID/g)
