# Supplementary material for: Non-invasive molecular imaging of inflammatory macrophages in allograft rejection
Source: EJNMMI Res. 2015 Nov 26;5:69. doi: 10.1186/s13550-015-0146-7 (PMC4661159; doi:10.1186/s13550-015-0146-7)
Supplement: Additional file 4: Table S2. — Biodistribution of 99mTc-IgG and 99mTc-SER-4 in C57Bl/6 wild-type (WT) and Sn-/- (KO) mice. Biodistribution of 99mTc-IgG isotype control in C57Bl/6 wild-type mice (WT × Isotype) 99mTc-SER-4 in C57Bl/6 wild-type (WT × 99mTc-SER4) and Sn-/- mice (Sn KO × 99mTcSER4) were expressed as percentage injected dose per gram of tissue (%ID/g). (PDF 70 kb) [file 13550_2015_146_MOESM4_ESM.pdf]

ESM Table 2

Alexander S. G. O'Neill[1,2], Samantha Y.A. Terry[1], Kathryn Brown[3], Lucy Meader[3], Andrew M.S. Wong[4], Jonathan D. Cooper[4], Paul R. Crocker[45], Wilson Wong[3], Gregory E. D. Mullen[1,3*]

[1]Department of Imaging Chemistry and Biology, Division of Imaging Sciences and Biomedical Engineering, King's College London, St. Thomas' Hospital, London, SE1 7EH, UK
[2]Division of Medical Sciences, University of Oxford, John Radcliffe Hospital, Oxford, OX3 9DU
[3]MRC Centre for Transplantation, King's College London, Guy's Hospital, London, UK
[4]Pediatric Storage Disorders Laboratory, Department of Neuroscience and Centre for the Cellular Basis of Behaviour, King's College London, London, UK
[5]Division of Cell Signalling and Immunology, College of Life Sciences, University of Dundee, Dundee, UK

	WT x isotype					WT x SER4					Sn KO x SER4				
Mouse	1.00	2.00	3.00	4.00	5.00	6.00	7.00	8.00	9.00	10.00	11.00	12.00	13.00	14.00	15.00
intestines	7.19	7.98	8.19	7.16	7.80	18.07	13.74	9.24	14.00	9.62	3.70	5.75	4.40	4.81	6.28
stomach	6.75	3.59	5.27	4.20	6.61	6.15	5.95	7.78	9.60	6.13	6.26	4.38	3.32	4.70	5.83
spleen	20.38	17.32	16.00	15.83	13.14	253.55	265.69	143.74	246.20	127.80	8.16	7.62	7.69	7.63	10.23
liver	24.57	27.74	24.73	22.06	32.70	41.76	48.49	30.01	39.50	32.22	8.87	10.56	8.42	12.18	10.03
kidney	29.58	34.86	35.76	30.99	32.07	30.98	21.51	25.85	24.86	26.32	13.95	20.40	15.76	22.45	18.31
heart	37.07	27.31	27.51	23.42	24.04	18.73	8.71	13.52	17.46	14.61	9.18	11.64	8.22	10.65	10.35
blood	49.08	57.35	42.98	54.51	60.67	30.53	19.41	27.59	31.28	21.49	26.54	36.42	29.29	49.31	25.87
lungs	28.98	29.34	29.39	27.16	25.79	17.06	8.76	17.49	11.44	13.23	16.22	17.64	14.66	15.46	16.61
muscle	3.60	4.31	3.57	3.31	2.74	12.98	1.28	1.54	1.67	1.87	2.21	3.14	2.37	2.19	2.74
bone	8.77	9.46	10.24	8.86	8.44	24.14	59.61	34.93	38.09	46.78	4.28	3.55	4.61	4.68	4.90

ESM Table 2. Biodistribution of ^{99m}Tc-IgG and ^{99m}Tc-SER-4 in C57Bl/6 wild type (WT) and Sn$^{-/-}$ (KO) mice. Biodistribution of ^{99m}Tc-IgG isotype control in C57Bl/6 wild type mice (WT x Isotype) ^{99m}Tc-SER-4 in C57Bl/6 wild type (WT x 99mTc-SER4) and Sn -/- mice (Sn KO x 99mTcSER4) were expressed as percentage injected dose per gram of tissue (%ID/g).
