# Supplementary material for: Non-invasive molecular imaging of inflammatory macrophages in allograft rejection
Source: EJNMMI Res. 2015 Nov 26;5:69. doi: 10.1186/s13550-015-0146-7 (PMC4661159; doi:10.1186/s13550-015-0146-7)
Supplement: Additional file 5: Table S3. — Biodistribution of 99mTc-SER-4 in heterotopic cardiac transplant model. Biodistribution of 99mTc-SER-4 in recipients of allogeneic heart graft (mouse 1–5), syngeneic heart grafts (mouse 6–9), or allogeneic heart grafts at 4 h post injection of 99mTc-IgG isotype control (mouse 10–14). Data expressed as percentage injected dose per gram of tissue (%ID/g). (PDF 67 kb) [file 13550_2015_146_MOESM5_ESM.pdf]

ESM Table 3

Alexander S. G. O'Neill[1,2], Samantha Y.A. Terry[1], Kathryn Brown[3], Lucy Meader[3], Andrew M.S. Wong[4], Jonathan D. Cooper[4], Paul R. Crocker[45], Wilson Wong[3], Gregory E. D. Mullen[1,3*]

[1]Department of Imaging Chemistry and Biology, Division of Imaging Sciences and Biomedical Engineering, King's College London, St. Thomas' Hospital, London, SE1 7EH, UK
[2]Division of Medical Sciences, University of Oxford, John Radcliffe Hospital, Oxford, OX3 9DU
[3]MRC Centre for Transplantation, King's College London, Guy's Hospital, London, UK
[4]Pediatric Storage Disorders Laboratory, Department of Neuroscience and Centre for the Cellular Basis of Behaviour, King's College London, London, UK
[5]Division of Cell Signalling and Immunology, College of Life Sciences, University of Dundee, Dundee, UK

	Allogeneic x SER4					Syngeneic x SER4				Allogeneic x isotype				
Mouse	1	2	3	4	5	6	7	8	9	10	11	12	13	14
intestines	2.37	3.10	2.93	1.87	3.21	6.30	5.20	6.48	8.07	9.59	10.69	7.82	6.94	5.55
stomach	2.18	3.31	2.12	2.38	3.52	3.98	2.97	3.80	7.62	4.81	4.16	5.25	5.98	4.95
spleen	48.01	47.32	50.14	52.31	66.78	72.15	85.26	128.98	113.47	8.68	7.98	9.26	8.46	7.77
liver	22.66	22.96	28.02	24.36	33.87	18.13	18.92	26.63	36.57	15.17	12.09	13.74	13.53	12.00
kidney	9.23	10.83	14.54	8.49	12.15	15.43	14.59	13.35	18.92	20.54	18.11	20.27	19.02	18.28
heart graft	7.70	8.83	7.21	9.37	14.10	4.57	4.20	4.59	3.99	4.99	6.03	5.90	6.38	6.38
heart native	2.35	2.84	2.51	2.91	5.42	3.99	4.28	11.74	20.51	14.26	15.75	17.39	18.60	15.05
lungs	2.46	3.10	2.58	3.10	3.73	4.83	4.00	4.18	5.90	11.95	10.53	11.94	13.03	9.65
blood	5.38	6.51	6.96	4.90	8.31	12.63	10.54	13.62	15.88	53.34	51.21	47.53	49.86	49.42
muscle	0.48	0.78	0.83	1.12	1.40	1.34	1.09	1.71	2.82	2.35	2.00	1.55	2.64	3.04
bone	18.00	19.54	23.23	10.41	28.53	23.11	28.71	23.02	24.87	5.78	7.94	6.55	4.55	4.76

ESM Table 3. Biodistribution of ^{99m}Tc-SER-4 in heterotopic cardiac transplant model. Biodistribution of ^{99m}Tc-SER-4 in recipients of allogeneic heart graft (mouse 1 – 5), syngeneic heart grafts (mouse 6 – 9), or allogeneic heart grafts at 4 hrs post injection of ^{99m}Tc-IgG isotype control (mouse 10 – 14). Data expressed as percentage injected dose per gram of tissue (%ID/g)
